# Fragment-Based Hologram QSAR Studies on a Series of 2,4-Dioxopyrimidine-1-Carboxamides As Highly Potent Inhibitors of Acid Ceramidase

**Published:** 2016

**Authors:** Xiang-Lin Yang, Yuan Zhou, Xin-Ling Liu

**Affiliations:** a *College of Chemistry and Chemical Engineering, Hunan University of Science and Technology, Xiangtan, Hunan 411201, China.*; b *College of Chemistry and Chemical Engineering, Hunan Institute of Engineering, Xiangtan, Hunan 411104, China.*

**Keywords:** Acid ceramidase, Drug design, Inhibitors, Hologram QSAR

## Abstract

A series of structurally related 2,4-dioxopyrimidine-1-carboxamide derivatives as highly potent inhibitors against acid ceramidase were subjected to hologram quantitative structure-activity relationship (HQSAR) analysis. A training set containing 24 compounds served to establish the HQSAR model. The best HQSAR model was generated using atoms, bond, connectivity, donor and acceptor as fragment distinction and 3–6 as fragment size with six components showing cross-validated q^2 ^value of 0.834 and conventional r^2^ value of 0.965. The model was then employed to predict the potency of test set compounds that were excluded in the training set, and a good agreement between the experimental and predicted values was observed exhibiting the powerful predictable capability of this model $\left(r_{\mathrm{pred}}^{2}=\mathrm{0.788}\right)$. Atom contribution maps indicate that the electron-withdrawing effects at position 5 of the uracil ring, the preferential acyl substitution at N3 position and the substitution of eight-carbon alkyl chain length at N1 position predominantly contribute to the inhibitory activity. Based upon these key structural features derived from atom contribution maps, we have designed novel inhibitors of acid ceramidase possessing better inhibitory activity.

## Introduction

Being essential structural elements of cell membranes, sphingosine-containing lipids serve significant signaling roles in the regulation of cell differentiation and growth, as well as in cell recognition, cell migration, and inﬂammation (1-3). The ceramides, a key member of the various groups of sphingolipids, have drawn particular attention for their functions in the replication and differentiation of tumor cells (4). The ceramide levels in some types of human tumors are much lower than in normal tissues, and the higher the ceramide levels, the lower the degree of malignant progression (5, 6). Moreover, various stress-related signals stimulate the generation of ceramide, which can in turn motivate the apoptosis of cancer cells (5, 6). Therefore, different interventions in the metabolic pathways of ceramide should inﬂuence cancer development and therapy. For instance, the recently reported 3-hydroxy vinylboronates trigger apoptosis in Jurkat cells by regulating sphingolipid metabolism resulting in the increase of the percentage of ceramide (7). Particularly, enzyme pathways implicated in regulating intracellular ceramide levels might emerge as potential new targets for antineoplastic therapy (8).

Acid ceramidase (AC) is a cysteine amidase that acts within the acidic conditions of the lysosome to hydrolyse ceramide into fatty acid and sphingosine (9). AC is not only associated with the control of ceramide levels in cells, but also regulates the ability of ceramide to affect the survival, growth and death of neoplastic cells (6-8). In line with this view, some types of human cancer (e.g. colon, head and neck, and prostate) express abnormally high levels of AC and serum AC levels are enhanced in melanoma patients compared with control subjects (10). Furthermore, over-expression of AC makes cells more resistant than normal cells to pharmacological induction of apoptosis, which is indicative of a role for this enzyme that inhibition of AC activity renders tumor cells more liable to the effects of chemotherapy and radiation (11, 12).

Despite the fact that several AC inhibitors, which can inhibit AC activity *in-vitro*, have been disclosed, potent compounds capable of inhibiting this enzyme *in-vivo* are still in demand, especially small-molecule compounds (13-15). The majority of AC inhibitors reported so far include oleoylethanolamide (OEA), D-MAPP, B-13, and their derivatives (16-21). Though useful experimentally, a common drawback of these inhibitors is that they are structurally related with ceramide, which leads to various limitations such as insufficient drug-likeness and inadequate activity *in-vivo* (8). For this reason, efforts have been made to discover new AC inhibitors with improved potency both *in-vitro* and *in-vivo*.

Recently, carmofur, which is employed to treat colorectal cancers in the clinic, were reported to be new AC inhibitors with nanomolar potency *in-vivo* (22). Further investigation demonstrated that carmofur can exert anti-proliferative effects because of the dominant inhibition of acid ceramidase. According to these findings, several derivatives, baesd on the chemical scaffold of carmofur, were synthesized and their inhibitory activity against acid ceramidase was also measured, leading to the discovery of new AC inhibitors (22, 23). Furthermore，preliminary structure−activity relationship (SAR) studies were also performed (23). However, rudimentary SAR studies often fail us to understand comprehensively the structural features required for the molecular biological activity, not to mention that SAR studies can be used to predict the biological activity of unsynthesized compounds. Thus, the findings obtained from SAR studies has some limitations with regard to providing guidelines for designing drug with enhanced biological activity.

Throughout recent years, fragment-based 2D QSAR methods served as versatile tools in drug design, among which hologram QSAR has emerged as a powerful strategy to investigate the chemical and biological properties for various types of compounds (24). Therefore, with the goal of identifying more potent *in-vivo* small-molecule inhibitors for acid ceramidase, we carried out hologram quantitative structure–activity relationship study for the series of structurally related 2, 4-dioxopyrimidine-1-carboxamide derivatives. On the basis of the HQSAR mode thus established, we attempted to elucidate a quantitative structure–activity relationship to provide useful guidelines for the design of more potent AC inhibitors.

## Experimental

*Data sets and Molecular modeling*

The inhibitory activity of the 2,4-dioxopyrimidine-1-carboxamide inhibitors of acid ceramidase (AC)，which has been reported by Pizzirani *et al*. (23), was taken for the study ( Table 1). The biological data taken from the literature as IC_50_ value of AC inhibition was converted to the corresponding pIC_50_ (-*log* IC_50_) and used as dependent variable in HQSAR analysis. The pIC_50_ values span a range of 4 log units, providing a broad and homogenous data set for the HQSAR study. Taking the structural diversities and wide range of activity into account, the compounds were divided randomly into training and test set. Meanwhile, a little care was taken in the selection of test set compounds, so that compounds in the training set were representative. Twenty-four of total 32 compounds were included in the training set to derive the HQSAR model while the remaining eight compounds were used as test set to validate the external predictability of the model. Molecular modeling studies were performed using the SYBYL 8.1.1 software package (Tripos, L.P., USA) running on a HP Z600 workstation. The molecular structures were sketched and minimized individually using Tripos force field. The minimum energy difference of 0.005 kcal/mol was set as a convergence criterion. 

**Table 1:** Chemical structures, experimental and predicted activities, and residuals of compounds included in the training set and test set.

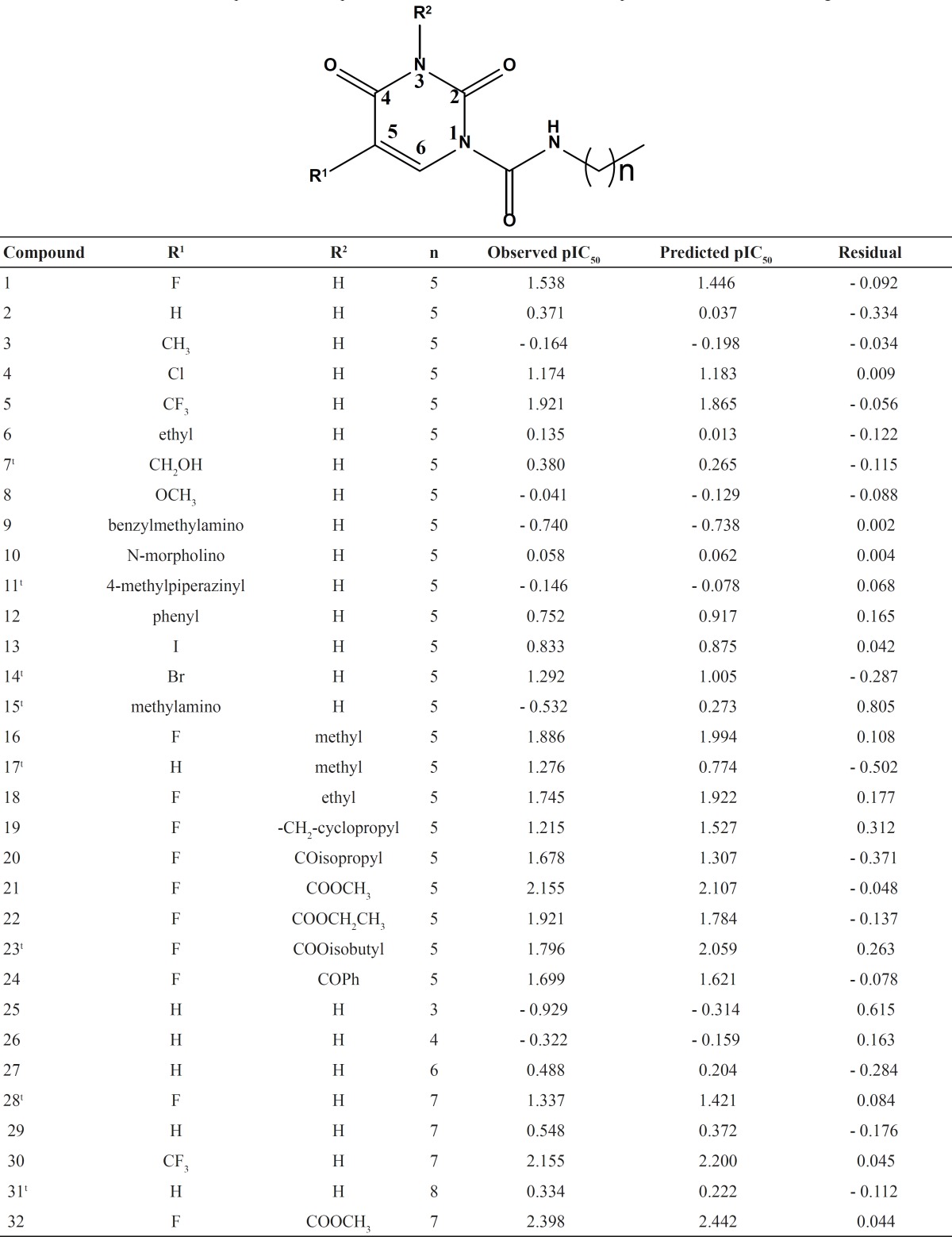

^t^ test set compounds

*Hologram QSAR method*

Hologram QSAR is a modern QSAR technique developed from unity hashed ﬁngerprint concept, which employs specialized fragment ﬁnger prints as predictive variables of biological activity (25). Compared with other existing methods for QSAR, HQSAR avoids not only the need for 3D structure, putative binding conformations, and molecular alignment in CoMFA (26) and CoMSIA (27), but also the selection and calculation or measurement of the physicochemical descriptors required by classical QSAR. HQSAR analysis involves three main steps: the generation of substructural fragments for each of the molecules in the training set; the encoding of these fragments into holograms; and correlation of molecular hologram with the available biological activity.

During hologram generation, the input molecule is broken into a series of unique structural fragments (linear, branched and overlapping) containing user-deﬁned minimum and maximum number of atoms. According to a predeﬁned set of rules that encodes the frequency of occurrence of various types of molecular fragment, the hashed ﬁngerprint is obtained. Then, this hashed ﬁngerprint is divided into strings at a ﬁxed interval as determined by a hologram length (HL) parameter. The strings are then aligned and the sum of each column constitutes the individual component of the molecular hologram of a particular length. 

A number of parameters concerning hologram generation, such as hologram length, fragment size and fragment distinction, prevailingly affect the HQSAR model quality (25). In order to derive the best HQSAR model, it is necessary to discuss the effects of various combinations of parameters on the HQSAR model. All models generated in these studies were evaluated using full cross-validated q^2^, partial least squares (PLS) and leave-one-out (LOO) method.

Predictive correlation coefficient $\left(r_{\mathrm{pred}}^{2}\right)$

The predictive ability of the HQSAR models was evaluated with predictive correlation coefficient $\left(r_{\mathrm{pred}}^{2}\right)$ deﬁned by Equation (1):

$\left(r_{\mathrm{pred}}^{2}\right)=\mathrm{SD}-\mathrm{PRESS})/\mathrm{SD}$

Equation (1)

Where SD is the sum of squared deviations between the biological activity of the test set compounds and the mean activity of the training set molecules, and the PRESS is the sum of squared deviations between predicted and observed activity values for every molecule in the test set.

## Results and Discussion

*HQSAR analysis for the effect of various fragment distinction combinations on the model quality*

For the sake of reducing the chances of bad collisions, the defaults of the hologram lengths are set automatically by software as several prime numbers, such as 53, 59, 61, 71, 83, 97, 151, 199, 257, 307, 353 and 401. Employing these prime numbers as hologram lengths, several combinations of these parameters were considered using the fragment size default (4–7) as follows: A/B, A/B/C, A/B/C/H, A/B/H, A/B/DA, A/B/C/DA, A/B/H/DA, A/B/C/H/DA. The fragment distinction parameters are described as follows: A, atoms; B, bonds; C, connections; H, hydrogen atoms; DA, donor and acceptor. Due to the lack of chiral carbon atom of all the 32 molecules, the fragment distinction of chirality was not discussed in Table 2. 

From what has been demonstrated in Table 2, we can obviously see that the best statistical model was derived using atoms, bonds, connections, donor and acceptor as fragment distinction with 6 being the optimum number of PLS components showing cross-validated q^2 ^value of 0.824 and conventional r^2^ value of 0.946. 

It is interesting to note that the statistical parameters in model 1 are equivalent to that in model 4 and the statistical parameters in model 2 are the same as that in model 3. The same phenomenon is observed between model 5 and model 7. Furthermore, there is not distinct difference in the statistical parameters between model 6 and model 8. Contrasting and analyzing the models mentioned above, we found one common feature that the latter model took into consideration an additional fragment distinction parameter, namely, hydrogen atoms, compared with the former model. In other words, the additional selection of hydrogen flag would do no good in ameliorating the model quality. In addition, obviously, the quality of model 1 that factored in atoms (A) and bonds (B) is relatively satisfactory. However, the additional selection of connections (C) or donor and acceptor (DA) led to a decrease in model quality, which can be verified from the statistical parameters of model 2 and model 5. Remarkably, the simultaneous introduction of connections (C) and donor and acceptor (DA) during the model building process on the basis of model 1 resulted in the best model (model 6), which may be due to the fact that C and DA played a synergetic role in enhancing the model quality. The synergistic action of connections (C) combined with donor and acceptor (DA) was also embodied between model 2 and model 6. Taken together, the important role of C and DA involved in developing the HQSAR model is indicative of the possibility that connections and donor and acceptor complement each other for the inhibitor-enzyme interaction, which should be still verified by experiment in future.

**Table 2:** HQSAR analysis for the effect of various fragment distinction combinations on the key statistical parameters using default fragment size (4-7

**Model**	**Fragment distinction**	**r** ^2^	**SEE**	**q** ^2^	**SEP**	**HL**	**N**
1	A/B	0.950	0.248	0.770	0.536	307	5
2	A/B/C	0.865	0.388	0.711	0.569	83	3
3	A/B/C/H	0.865	0.388	0.711	0.569	83	3
4	A/B/H	0.950	0.248	0.770	0.536	307	5
5	A/B/DA	0.914	0.318	0.767	0.524	353	4
6	A/B/C/DA	0.946	0.267	0.824	0.481	257	6
7	A/B/H/DA	0.914	0.318	0.767	0.524	353	4
8	A/B/C/H/DA	0.946	0.267	0.824	0.481	257	6

q^2^, cross-validated correlation coefficient; SEP, cross-validated standard error; r^2^, noncross-validated correlation coeﬃcient; SEE, non cross-validated standard error; HL, hologram length; N, optimal number of components. Fragment distinction: A, atoms; B, bonds; C, connections; H, hydrogen atoms; DA, donor and acceptor.

The model chosen for analysis is highlighted in bold fonts.

*HQSAR analysis for the inﬂuence of various fragment size on model quality*

Based on the best HQSAR model generated above (model 6, Table 2), the influence of different fragment sizes on statistical parameters was further investigated and summarized in Table 3. As can be seen from Table 3, the r^2^ values of all models are greater than 0.89, and the q^2 ^values are also satisfactory. The results shown in bold fonts in table 3 indicated that the fragment size（3-6）led to better statistical results in comparison with other fragment sizes. Therefore, the best final HQSAR model obtained from training set with 24 compounds was established using atoms, bonds, connections, donor and acceptor as fragment distinction and 3-6 as fragment size with 6 being the optimum number of PLS components showing cross-validated q^2 ^value of 0.834 and conventional r^2^ value of 0.965.

**Table 3:** HQSAR analysis for the inﬂuence of various fragment size using the best fragment distinction (A/B/C/DA). The model chosen for analysis is highlighted in bold fonts

**Fragment size**	**r** ^2^	**SEE**	**q** ^2^	**SEP**	**HL**	**N**
1-3	0.892	0.348	0.725	0.555	401	3
4-7	0.946	0.267	0.824	0.481	257	6
3-10	0.933	0.298	0.721	0.606	353	6
1-4	0.912	0.315	0.781	0.495	83	3
2-5	0.934	0.272	0.807	0.466	151	3
3-6	0.965	0.214	0.834	0.468	257	6
5-8	0.939	0.283	0.762	0.560	353	6
6-9	0.934	0.295	0.740	0.585	353	6
7-10	0.927	0.309	0.736	0.589	353	6

*The evaluation of HQSAR model quality*

**Figure 1:**
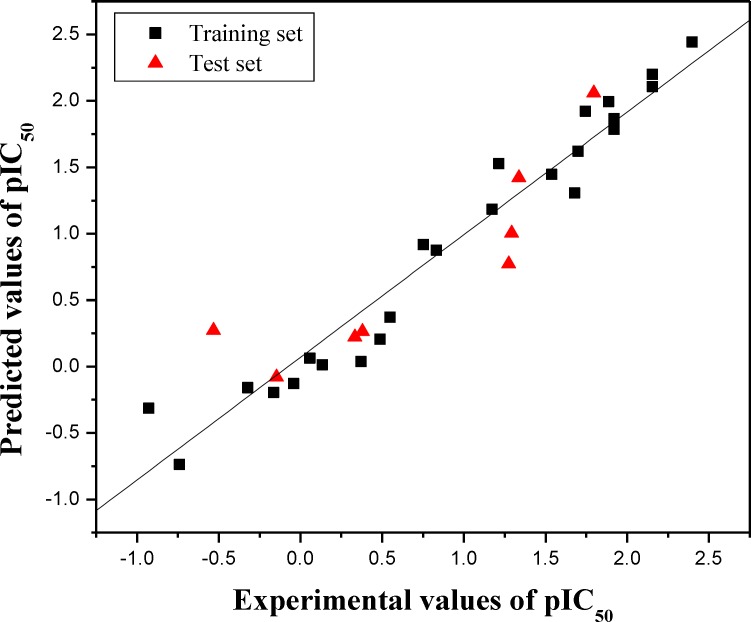
Plot of experimental versus predicted pIC_50_ values of the training set and test set molecules

Since the structure encoded within a 2D ﬁngerprint is directly related to biological activity of molecules, the HQSAR model is able to predict the activity of structurally related molecules according to its ﬁngerprint. In virtue of the finally accepted QSAR model showing non-cross-validated (r^2^ =0.965) and cross-validated (q^2 ^=0.834) correlation coefficients, which manifested a good internally predictive power, the predicted pIC_50_ values of both test set and training set compounds are listed in Table 1. Furthermore, the graphic results for the experimental versus predicted activities of both training set and test set are displayed in Figure 1. The constructed HQSAR model has good agreement between experimental and predicted values for the test set compounds with the higher predictive correlation coefﬁcient $\left(r_{\mathrm{pred}}^{2}\right)$(= 0.788), which signified a high external predictability of model. As far as the satisfactory performance of this holographic QSAR is considered, the model can be used to predict the biological activity of novel compounds within this structural class. 

The training set and test set molecules are shown in black (squares) and red (triangle) spots, respectively.

*Interpretation of HQSAR contribution map*

A significant role of a QSAR model is not only to predict the activities of untested molecules, but also to throw light on what molecular fragments play key roles to the contribution of biological activity. The results of the HQSAR analysis is graphically displayed as a color-coded structure diagram in which the color of each atom reﬂects the contribution of that atom to the molecule’s overall activity. The colors at the red end of the spectrum (red and orange) represent poor contributions, while colors at the green end (yellow, blue and green) indicate favorable contributions. HQSAR offers a good way of accounting for the variance of molecular activity by condensing information on the structural fragment. 

**Figure 2:**
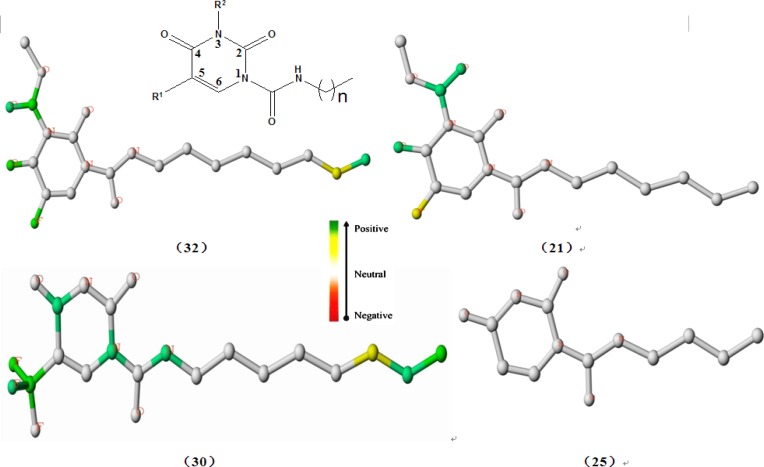
Atomic contribution maps for compounds 32, 30, 21 and compound 25

Using the best HQSAR model, which factored atoms, bonds, connections; donor and acceptor into fragment distinction parameters, the atomic contribution maps of 24 compounds included in the training set were generated. The individual atomic contribution maps of the first single-digit potent nanomolar acid ceramidase inhibitors（compound 32, 30 and 21）as well as the least potent AC inhibitor (compound 25), resulting from the best HQSAR model, are displayed in Figure 2. As known to us, the different substituents with various chemical properties attached to the 2,4-dioxopyrimidine-1-carboxamide scaffold incurred different responses to the inhibition of AC activity，which is especially embodied at the position N3 and N5 of the uracil ring in addition to the alkyl side chain at N1 position (23).

First of all, it can be seen obviously from Figure 2 that the individual atomic contribution map of compound 25 is colored white totally because it serves as the common structure that exists in every studied molecule. 

With respect to the impact of R_1_ substituent on the inhibition of AC, the fluorine atoms tethered to the position N5 of the uracil ring both in the compound 32 and 21 were colored green and yellow respectively, indicating its positive contribution to inhibitory activity, which explained well why compounds 1, 16, 28 have higher potency than compounds 2, 17, 29. Furthermore, the trifluoromethyl group in the same place (compound 30) was colored heavily green, signifying its highly beneficial contribution to inhibitory activity, which is a possible reason why compound 30 has higher potency than compound 29. In consideration of the preeminent role of the fluorine atom and the trifluoromethyl group at N5 position of the uracil ring, it can be deduced that the introduction of electron-withdrawing group will play a crucial role in improving the inhibitory activity of this class of compounds, which was borne in mind in our follow-up molecular design. This conclusion is also consistent with previous SAR studies, which reinforced the importance of electron-withdrawing effect in enhancing the AC inhibitory activity (23). On the other hand, in combination with the above-mentioned analysis about the role of connections (C) and donor and acceptor (DA) in developing the HQSAR model, we come up with the presumption that the electron-withdrawing group (R_1_) such as fluorine atom or trifluoromethyl may act as hydrogen bond acceptor for the inhibitor-enzyme interaction, as proposed in the discussion about Table1.

As regards the influence of alkyl chain length on the inhibition activity of AC, the carbon atoms at the tip of the chain in compound 32 and compound 30 were colored yellow or green while the terminal atoms in compound 21 and compound 25 were colored white, which provided a hint that compounds bearing eight-carbon alkyl chain exhibits higher AC inhibition activity than compounds with other alkyl chain length. In other words, eight-carbon alkyl chain length is superior to other alkyl chain length for improving the AC inhibition activity, as also evidenced by the higher predicted pIC_50_ values of the designed molecules (E) compared with compounds (C and D), which may be the very reason why compound 32 possesses higher AC inhibition activity than compound 21.

In addition, of particular interest was the green color of the 1-carboxamide NH group in compound 30, implying its favorable contribution to the AC inhibition activity, which also shed light on the key role of 1-carboxamide NH moiety essential for the AC inhibition activity of this class of compounds (23). 

Furthermore, what interested us was that the oxygen atoms or carbon-oxygen double bond located at position 4 of the uracil ring were all colored green in compounds 32, 30, 21. Although these fragments are a part of the common structure incorporated in all the studied molecules, they seemed to provide some hints about the key function of a fully conjugated 2,4-dioxopyrimidine-1-carboxamide system, as verified by the computational studies (23). On the other hand, the oxygen atoms may function as hydrogen bond acceptor for the inhibitor-enzyme interaction, as put forward in the analysis for Table 1, which is a hypothesis needing to be confirmed in further investigation.

*Designed compounds and predicted activity*

**Figure 3:**
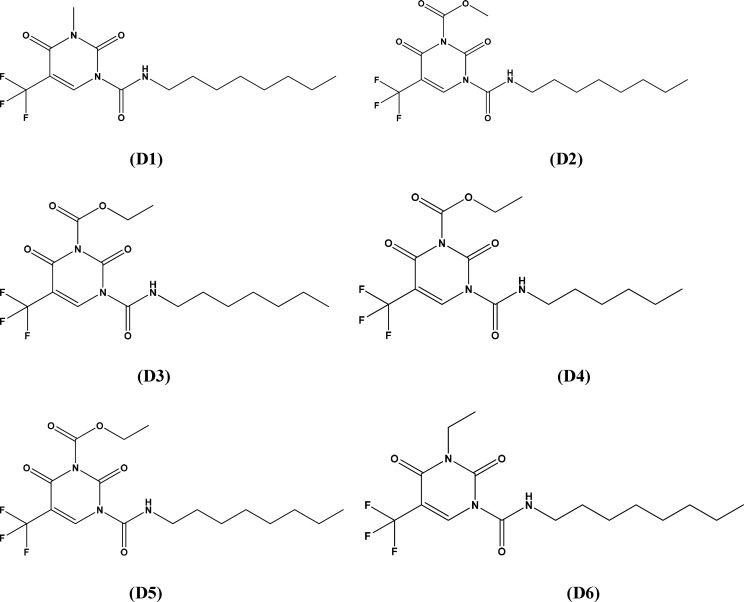
Structures of designed compounds with potentially improved biological activity

In terms of the information derived from these contribution maps together with the analysis thus made above, we further modified the structure of 2,4-dioxopyrimidine-1-carboxamide acid ceramidase inhibitors. The structures of new compounds with potentially improved biological activity were displayed in Figure 3. Taking advantage of the best holographic QSAR model established above, the activities of the new compounds thus designed were predicted, as shown in Table 4. According to the prediction results, the biological activities (p*IC*_50_) of new compounds were all greater than 2.4. These new compounds are likely to possess higher inhibitory activity, which remains to be experimentally verified.

**Table 4:** Chemical structures of designed molecules and predicted biological activities

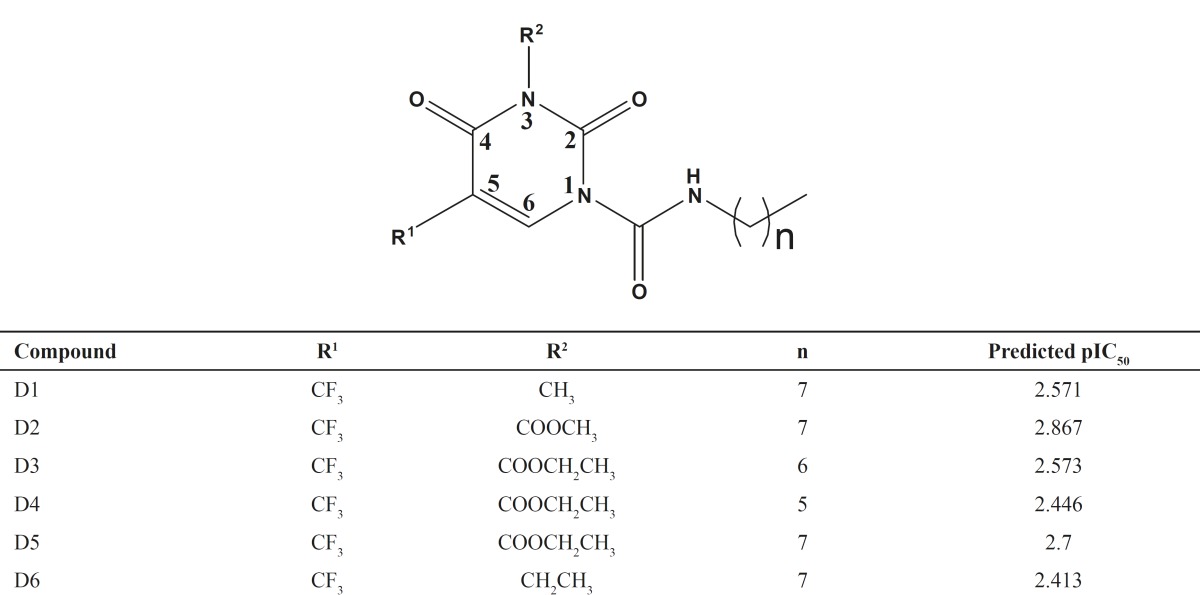

## Conclusions

In summary, we successfully generated a hologram QSAR model for the 2,4-dioxopyrimidine-1-carboxamides as acid ceramidase inhibitors with good statistical results. The model (N= 6) displayed significant cross-validated (q^2^= 0.834) and non-cross-validated correlation coefficients (r^2^= 0.965). The strong agreement between the experimental and predicted values for the test set compounds verified the reliability and robustness of the constructed HQSAR model, indicating a high external predictability of model ($\left(r_{\mathrm{pred}}^{2}\right)$= 0.788). The contribution of the structural fragment to the biological activity of this series of compounds was interpreted by the HQSAR contribution maps. Atom contribution maps suggested that the electron-withdrawing effects at position 5 of the uracil ring, the preferential acyl substitution at position N3 and the eight-carbon alkyl chain length at N1 position dominantly increased the inhibitory activity. Finally, based on the findings mentioned above, we have designed novel inhibitors of acid ceramidase possessing better inhibitory activity. Therefore, the HQSAR model can provide guidelines for future efforts in the design of new more active AC inhibitors that are structurally related with the training set compounds.
